# Integrated Risk Information System (IRIS) response to “Assessing risk of bias in human environmental epidemiology studies using three tools: different conclusions from different tools”

**DOI:** 10.1186/s13643-021-01783-6

**Published:** 2021-08-21

**Authors:** Elizabeth G. Radke, Barbara S. Glenn, Andrew D. Kraft

**Affiliations:** Center for Public Health and Environmental Assessment, Office of Research and Development, U.S. Environmental Protection Agency, Washington, D.C, USA

**Keywords:** Systematic review, Environmental epidemiology, Risk of bias, Study evaluation, Risk assessment

## Abstract

“Assessing risk of bias in human environmental epidemiology studies using three tools: different conclusions from different tools,” a recent publication in this journal, applied the study evaluation approach developed by the U.S. Environmental Protection Agency’s Integrated Risk Information System (IRIS), as well as other approaches, to a set of studies examining polybrominated diphenyl ethers (PBDEs) and neurodevelopment. They concluded that use of the IRIS approach resulted in exclusion of studies, which would lead to hazard conclusions based on an incomplete body of evidence. As scientists in the IRIS program, we support the comparison of approaches to improve systematic review methods for environmental exposures; however, we believe the IRIS approach was misrepresented. In this letter, we demonstrate that the ratings attributed to the IRIS approach were not consistent with our own application of the tool. We also clarify the use of studies rated as “low confidence” and the use of an overall study confidence rating in our systematic reviews. In conclusion, the IRIS study evaluation approach is a transparent method to inform certainty in our evidence synthesis decisions and ensures consistency in the development of IRIS health assessments.

“Assessing risk of bias in human environmental epidemiology studies using three tools: different conclusions from different tools” by Eick et al. [1] applied the study evaluation approach developed by the U.S. Environmental Protection Agency’s Integrated Risk Information System (IRIS), as well as other approaches, to a set of studies examining polybrominated diphenyl ethers (PBDEs) and neurodevelopment. They concluded that use of the IRIS approach resulted in excessive exclusion of studies, which would lead to hazard conclusions based on an incomplete body of evidence.

As scientists in the IRIS program, we support the comparison of approaches to improve systematic review methods for environmental exposures; however, we believe the IRIS approach was misrepresented. While there is not space in this letter to describe the IRIS approach, we encourage readers to review our detailed methods for study evaluation in the ORD Staff Handbook for Developing IRIS Assessments [2]. This was recently publicly released and is undergoing peer review by the National Academies of Sciences, Engineering, and Medicine (https://www.nationalacademies.org/our-work/review-of-epas-iris-assessment-handbook). Examples of the actual application of these methods by the U.S. EPA are available in several systematic reviews of the health effects of phthalate exposure [3] and the recent assessment of perfluorobutane sulfonic acid [4]. In this letter, we make the following clarifications.

First, Eick et al. [1] reported that the IRIS study evaluation approach resulted in low confidence or uninformative ratings in all studies; however, we did not reach the same conclusions with our analysis of the same studies. As described in the IRIS Handbook, the study evaluation process begins with development and pilot testing exposure- and outcome-specific criteria that identify the information and appropriate methods needed to apply the evaluation ratings in each domain (for examples, see [5]). These criteria are based on the state of knowledge about the toxicokinetics of the chemical being assessed, exposure assessment methods, and the epidemiological standard of practice for specific outcomes. They improve transparency and consistency of our reviews, and were not part of the Eick et al. [1] evaluation. Importantly, in addition to risk of bias, the IRIS study evaluation approach considers the sensitivity of a study to detect effects if they exist, which improves our ability to interpret study findings. To illustrate how the approach is used by IRIS scientists, we re-applied the IRIS tool to the same 10 studies of PBDE exposure and IQ as in Eick et al. [1].

The results of our evaluation are presented in Fig. 1, and the rationales for each rating are available at https://hawcprd.epa.gov/summary/visual/100500646/. In the application of the IRIS approach by Eick et al. [1], the majority of studies were rated as “deficient” for confounding (8/10) and participant selection (7/10). For overall confidence, nine were low confidence and one was uninformative. In contrast, our evaluation identified 3/10 studies as “deficient” in confounding and participant selection, as well as low confidence overall. The remaining studies were rated as high (1) or medium (6) confidence overall. None were rated as “uninformative,” the judgment that would be necessary for study exclusion using the IRIS approach.

**Fig. 1:**
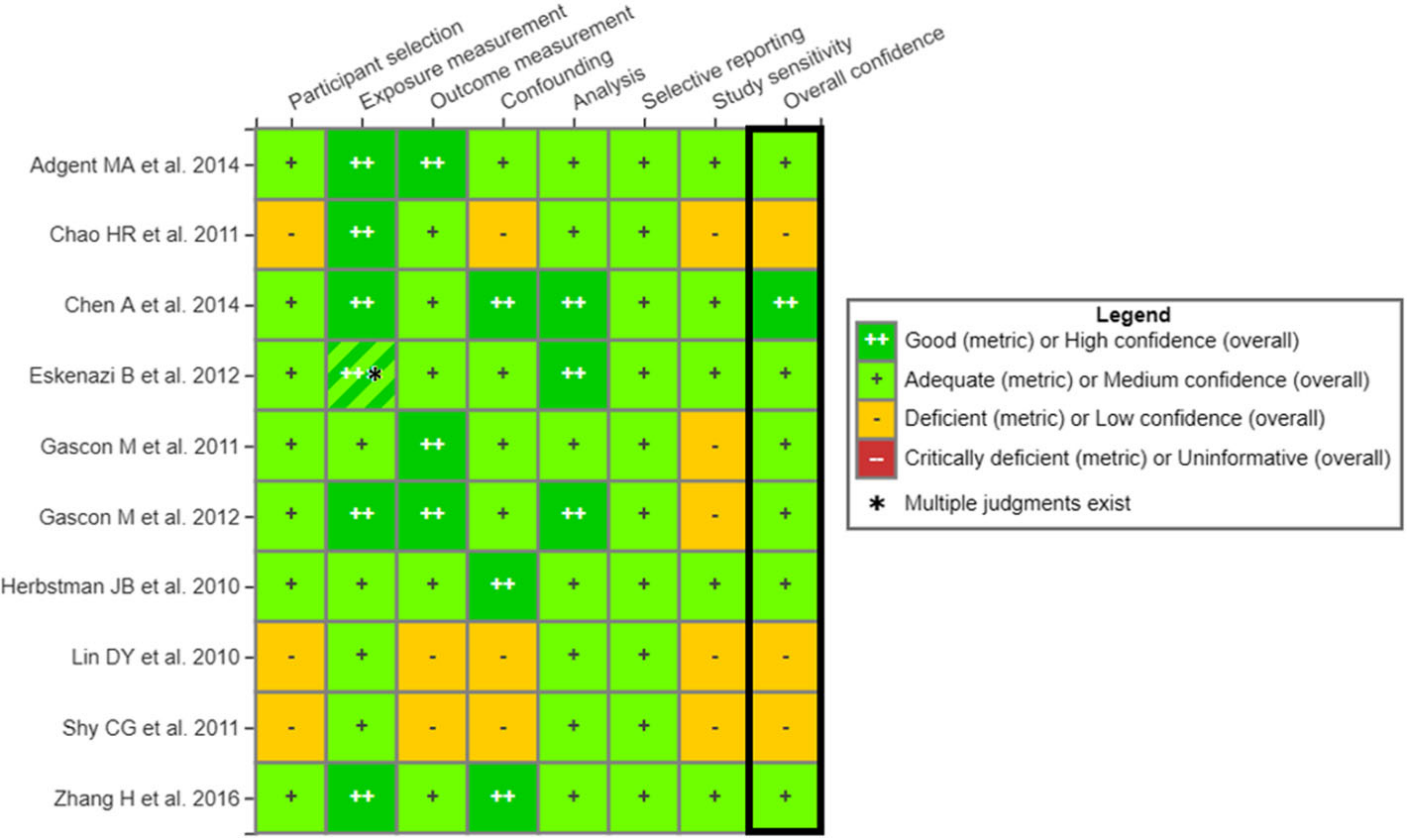
Heat map of re-evaluations of studies of PBDE exposure and IQ using Integrated Risk Information System (IRIS) approach. Evaluation rationales are available at: https://hawcprd.epa.gov/summary/visual/100500646/. Hashed shading indicates that there are multiple judgments in this domain for the study, which analyzed associations with exposure metrics with different temporal relationships with the outcome

Second, Eick et al. [1] states that in their case study, studies deemed “low” confidence or “uninformative” overall would be removed from the overall body of evidence. This decision is not consistent with our published methods, including the IRIS Handbook, which state that “Low confidence results are given less weight compared to high or medium confidence results during evidence synthesis and integration” [2]. Low confidence studies are included in the evidence synthesis, and comparisons of these results with those of high or medium confidence studies facilitate the review of consistency (i.e., between-study heterogeneity). Since low confidence studies have deficiencies expected to have a notable impact on the results, findings reported in these studies are less certain and considered with far more caution during evidence synthesis. Uninformative studies, on the other hand, are excluded from further evidence synthesis, consistent with the practices of NTP RoC [6] and ROBINS-I [7], because the evaluation found “serious flaw(s) [that] make the study results unusable for informing hazard identification” [2]. Since we did not identify any uninformative studies in our evaluations of the IQ studies, all would be included in an evidence synthesis.

Third, the analysis by Eick et al. [1] also objects to the use of an overall study rating. The IRIS approach includes a qualitative study confidence rating that considers the strengths and limitations identified in the individual domains and is based on expert judgment of the likely impact of the specific identified deficiencies on the individual study results. There is explicitly not a weighting of domains or quantitative scheme for reaching these overall ratings; one impactful limitation or a combination of identified deficiencies can result in a rating of low confidence. The analysis by Eick et al. [1] acknowledges that there is flexibility in the application of the overall study confidence rating, but incorrectly presents it as an override of what they interpret as a more deterministic approach where the number of *good*, *adequate*, and *deficient* ratings are counted to obtain an overall rating. Eick et al. [1] pointed to several references to support their arguments against overall quality scores; however, these were discussions of the limitations of *quantitative scoring*, which is not an approach we advocate or use. In contrast to the direction chosen by the Navigation Guide contributors, other institutions have adopted a strategy similar to the IRIS approach of reaching an overall qualitative rating or conducting stratified analyses based on the risk of bias/quality judgments [6–8]. The IRIS overall study confidence ratings are documented and presented with the individual domain ratings and their underlying rationale. The overall study confidence ratings in IRIS are always used and interpreted with that context. These ratings primarily help to focus the synthesis of evidence, allowing for stratification of results by overall confidence (in addition to by domain ratings if relevant) and increasing transparency. This is similar to the suggestion by Eick et al. of stratifying results in meta-analyses based on overall study quality. Overall confidence ratings also facilitate transparency in the process of selecting study results to analyze dose-response. If a hazard is identified by IRIS, then dose-response analyses are typically pursued for medium and high confidence studies.

In conclusion, the IRIS study evaluation approach is a transparent method to inform certainty in our evidence synthesis decisions and ensures consistency in the development of IRIS health assessments.

## Data Availability

Not applicable.

## Abbreviations

IRIS: Integrated Risk Information System
ORD: Office of Research and Development
EPA: Environmental Protection Agency
PBDEs: Polybrominated diphenyl ethers

## References

[1] Eick SM, Goin DE, Chartres N, Lam J, Woodruff TJ (2020). Assessing risk of bias in human environmental epidemiology studies using three tools: different conclusions from different tools. Syst Rev.

[2] U.S. EPA (2020). ORD Staff Handbook for Developing IRIS Assessments (Public Comment Draft, Nov 2020).

[3] Radke EG, Yost EE, Roth N, Sathyanarayana S, Whaley P (2020). Application of US EPA IRIS systematic review methods to the health effects of phthalates: lessons learned and path forward. Environ Int.

[4] U.S. EPA (2021). Human health toxicity values for perfluorobutane sulfonic acid and related compound potassium perfluorobutane sulfonate.

[5] Radke EG, Glenn BS, Galizia A, Persad A, Nachman R, Bateson T, Wright JM, Navas-Ascien A, Arroyave WD, Puett RC, Harville EW, Pollack AZ, Burns JS, Lynch CD, Sagiv SK, Stein C, Cooper GS (2019). Development of outcome-specific criteria for study evaluation in systematic reviews of epidemiology studies. Environ Int.

[6] National Toxicology Program (2015). Handbook for Preparing Report on Carcinogens Monographs.

[7] Sterne JA, Hernán MA, Reeves BC, Savović J, Berkman ND, Viswanathan M, Henry D, Altman DG, Ansari MT, Boutron I, Carpenter JR, Chan AW, Churchill R, Deeks JJ, Hróbjartsson A, Kirkham J, Jüni P, Loke YK, Pigott TD, Ramsay CR, Regidor D, Rothstein HR, Sandhu L, Santaguida PL, Schünemann HJ, Shea B, Shrier I, Tugwell P, Turner L, Valentine JC, Waddington H, Waters E, Wells GA, Whiting PF, Higgins JP (2016). ROBINS-I: a tool for assessing risk of bias in non-randomised studies of interventions. BMJ.

[8] National Toxicology Program (2019). Handbook for Conducting a Literature-Based Health Assessment Using OHAT Approach for Systematic Review and Evidence Integration.

